# Supplementary material for: Are carbon emissions trading and green financial instruments synergistic? -Comprehensive quantitative research based on content analysis
Source: PLoS One. 2024 Mar 7;19(3):e0298601. doi: 10.1371/journal.pone.0298601 (PMC10919601; doi:10.1371/journal.pone.0298601)
Supplement: S1 Data — (ZIP) [file pone.0298601.s001.zip › Data/地级市Z_F工作报告.zip!/╣π╬≈/║╪╓▌╩╨/└┤╫╘╣1⁄2╓┌║┼í╛┬φ┐╦╩2╛▌═°í┐_2012.docx]

各位代表:

　　 现在,我代表市人民政府向大会作工作报告,请予审议,并请市政协委员和其他列席人员提出意见。

一、2011年工作回顾

　　 过去的一年,面对极其复杂的内外环境和极其繁重的改革发展任务,我们在自治区党委、政府和市委的正确领导下,在市人大、市政协的监督支持下,团结依靠全市各族人民,深入贯彻落实科学发展观,集中力量“保投入、保扩量、保速度、保质量、保控价、保进位”,稳步推进各项工作,完成了市二届人大八次会议确定的目标任务,全市经济社会呈现出“发展加快、效益提升、活力增强、物价回落、民生改善”的良好态势,实现了“十二五”时期良好开局。

　　 初步预计,全市完成地区生产总值350亿元,增长13%；财政收入26.6亿元,增长20.7%；全社会固定资产投资463亿元,增长28%；规模以上工业总产值240亿元,增长47.0%；规模以上工业增加值80亿元,增长30%；社会消费品零售总额92亿元,增长17.5%；外贸进出口总额1.45亿美元,增长38.7%；城镇居民人均可支配收入17600元,增长11.5%；农民人均纯收入4900元,增长12%；城镇登记失业率为3.56%,人口自然增长率为8.14‰。总体上看,主要指标均完成或超额完成年度计划任务。预计规模以上工业总产值、规模以上工业增加值、外贸进出口总额等多项经济指标增幅在全区排位居前列；经济结构进一步优化,第二产业占地区生产总值的比重首次突破50%,工业主导地位得到巩固提高。

　　 一年来,我们主要做了以下工作:

　　 （一）着力破解发展难题。面对各种严峻形势,我们千方百计破解发展中遇到的“缺钱、缺地、缺电”和物价高位运行等突出难题,确保各项目标任务完成。加强财税工作,财政收入总量比上年净增4.5亿元,其中税收收入占财政收入的比重高达81.9%。深化政企银合作,2011年12月末全市金融机构存款余额298.90亿元,比年初增长18.75%,在全区排第1位；人民币各项贷款余额173.76亿元,比年初增长20.25%,在全区排第5位,余额存贷比达到58.13%；落实中小企业信贷资金10.61亿元,为140家中小企业担保贷款6.33亿元。加大向上级汇报衔接力度,争取国家投资项目322项,获资金7.21亿元。全力推进760公顷城区征地拆迁任务,争取用地指标545.14公顷,盘活供应存量建设用地507.94公顷。保障全社会用电量45.4亿千瓦时,其中工业用电量36.4亿千瓦时,在电力电量双缺情况下实现了企业产量和效益全面提升,规模以上工业产品产销率达到96%,实现利润增长55%。投入控价补贴财政资金544.5万元,启动限价大米、猪肉、食用油等重点商品销售工作,居民消费价格指数涨幅由7月份最高的9.4%下降到12月份的6.7%；建立社会救助和保障标准与价格上涨联动机制,落实补贴资金3176.9万元,惠及19万城镇低保人员、农村低保人群和五保对象。

　 　（二）着力推进项目建设。全市建设项目2241个,投产项目1456个,固定资产投资增量连续三年保持在100亿元左右,对保持经济平稳较快发展起到了决定性作用。完成市“十二五”规划主要建设项目计划编制。为确保完成全年投资任务,对全市828项投资额1000万元以上的主要项目进行重点管理,其中市级层面统筹推进重点项目128项,完成投资128亿元；自治区层面统筹推进重点项目29项,完成投资40.95亿元。以非常之举强化项目推进工作机制,推动翔隆电子、高峰桂山人造板等一批项目开工建设,华润贺州电厂、桂东电子技改等一批续建重大项目加快推进,华桂林浆纸一体化、贺州机场等一批项目前期工作有序推进。

　　 （三）着力壮大支柱产业。预计全市完成工业总产值380亿元,增长36.0%；工业增加值141亿元,增长19.4%。加快传统产业改造升级步伐,完成技改投资100亿元,增长32.3%。加快发展战略性新兴产业,编制实施电子信息、新材料、稀土产业发展规划,新材料产业工业总产值增长168%。实施中小企业成长工程,新增规模以上工业企业20家,新增中小企业66家。电力、林产、矿业、电子和新材料五大支柱产业实现工业总产值169.7亿元,增长45.6%,占规模以上工业总产值的78%。完善主要园区招商引资政策,完成新建园区规划编制,六大园区完成基础设施和产业项目投资61.7亿元,全市工业园区总产值增长43%,规划新建稀土产业园、钨钛锡新材料产业园、石材粉体产业园。规划建设贺州至富川一级公路,加快构建百里工业走廊。

　　 （四）着力改善发展环境。深入开展城市建设大会战,太白湖公园、绿洲家园、农民回建安居工程、传媒中心,以及灵峰大桥、西南环路改造工程、城东新区路网等城市路网工程开工建设,城建项目完成投资75.7亿元。进一步完善县城基础设施,深入实施14个重点乡镇风貌改造工程,加快建设黄姚镇、秀水村和福溪村等广西特色名镇名村,全市城镇化率达到37%,比上年提高2个百分点。推进104个新农村示范点建设,新建农村沼气池1.2万座,新建或改造农村公路282公里、农村电网线路480.3公里。加快推进贵广高速铁路贺州段、贺州至姑婆山公路、八步经黄姚至昭平二级公路二期等项目建设,永贺高速公路贺州段大岭头隧道、富江1号大桥等6个控制性工程进入实质性建设阶段,交通项目完成投资33亿元。加快建设上程水电站等电源、电网项目,全面实施农村饮水安全保障、中小河流治理、病险水库除险加固和农田水利建设“四大工程”,完成水利电力项目投资29亿元。大力实施“绿满贺州”工程,完成山上造林24.8万亩、通道绿化52公里、城镇绿化443亩。市本级污水处理率达到80%,县区污水处理率达到65%,城区生活垃圾无害化处理率达到100%。抓好重点企业节能技改工程,落实了“十二五”污染物总量控制年度指标,完成了年度计划减排项目。

　 （五）着力发展现代农业。完成农林牧渔业总产值120亿元,增长5.2%。实施万元增收工程、“234”产业化工程、三大增粮增收行动,农机总动力达到105万千瓦,发展万元片区15个、万元村屯22个、年纯收入10万元以上的致富示范户120户,粮食总产量达69.95万吨。全面推广以沼气为纽带的循环农业发展模式,新建生态循环农业示范村3个、生态文明示范村12个；通过无公害农产品产地认定种植面积达98.3万亩,创建出口农产品备案基地18个,富川率先建设成为广西唯一的国家级出口食品农产品质量安全示范区,平桂、八步、昭平获得广西出口食品农产品质量安全示范区称号。加快农业产业化步伐,成功引进雨润集团建设200万头生猪加工项目；嘉宝食品、广东温氏、富川杨氏果业等一批农业产业化项目加快推进；建成标准化农作物种植基地1909个、标准化规模养殖示范场141个,市级以上重点农业产业化龙头企业达40家,自治区级重点龙头企业6家。

　　 （六）着力发展商贸旅游业。精心打造姑婆山国家森林公园、黄姚古镇等重点景区景点,有序推进贺州大酒店、姑婆山客家风情小镇等项目建设,积极拓展旅游客源市场,接待游客总人数达662.6万人次,增长31.5%；实现旅游总收入54.5亿元,增长44.5%。加快商贸物流业发展,实施“万村千乡”市场工程,稳步推进湖广大市场、远东国际城等商贸项目建设。有效促进城乡消费,住宅销售额增长20.4%；家电下乡产品销售额达3.42亿元,增长52.9%。

　　 （七）着力深化改革开放。加快推进扩权强县进程,平桂管理区列入全国第三批资源枯竭城市。集体林权制度主体改革工作高质量通过自治区验收。新成立市矿业投资公司,中行贺州支行升格为二级分行,农村金融改革试点工作进展顺利,东莞农村商业银行在我市设立村镇银行工作顺利推进。推进粤桂合作贺州-佛山开发试验区规划建设。成功举办2011年桂东承接产业转移示范区建设联席会议,示范区建设上升为国家战略。成功举办第八届中国-东盟博览会贺州专场项目签约会、首届广西园博会贺州园、第六届广西贺州农展会等系列会展活动,多次组团到国内发达地区和台湾开展文化经贸洽谈活动。深化与华润集团、中国大唐、中国纸业、中国铝业、中国有色矿业、中国通用技术、中铁十六局等央企,以及广西交通投资集团、北部湾国际港务集团等自治区重点企业合作,开工建设华润雪花啤酒(广西)项目、龙头风电场等项目,达成了新材料、旅游、基础设施等一批项目投资意向。全年共引进外来投资项目510个,新签项目投资总额270亿元；内资项目到位资金280亿元,增长10.3%；外资项目到位资金8927万美元,增长26.9%。

　　 （八）着力保障改善民生。进一步完善“小财政办大民生”工作机制,持续加大对重点民生领域的财政投入,其中:农林水事务支出增长17.9%,住房保障支出增长57.6%,医疗卫生支出增长65.5%。顺利实施农田水利建设、贫困村道路建设、社会保障惠民、巩固和完善新型农村合作医疗制度、村级公共服务中心建设、沼气池建设、安居惠民、市幼儿园搬迁建设、城市管道燃气、市职教中心建设等10项为民办实事工程。城镇新增就业1.59万人,下岗失业人员实现再就业4385人；农业劳动力转移新增就业4.02万人。养老、医疗、失业、工伤、生育五项社会保险新增扩面1.54万人次,新农合参合率提高到94.34%。新开工建设保障性安居工程9328套,农村危房改造1.84万户。顺利通过第三批整村推进贫困村扶贫开发工作验收。全面恢复设立乡镇民政机构,把民政服务网络延伸到村(社区)一级。深入实施第五轮创新计划,专利申请量增长79.72%,授权量增长33.33%。投入3.8亿元推进贺州高中迁建工程和贺州二高扩建工程,如期完成市幼儿园、市职教中心建设任务,获全区职业教育攻坚进步市称号。加快构建覆盖城乡的公共文化服务体系,广播综合覆盖率达到94%,电视综合覆盖率达到98.1%,瑶族鼓舞鼓乐《鼓动瑶山》获第十届中国民间文艺山花奖·民间艺术表演一等奖。医疗卫生体制改革实现全市所有公办的基层医疗卫生机构全覆盖,新开工建设或建成村标准化卫生室707个。在全区第十二届运动会上获得45枚金牌,居全区第6位。人口计生综合改革全面深化,获自治区推荐为全国人口计生综合改革示范市。食品药品安全工作进一步加强。安全生产形势平稳。再获“全区双拥模范城”荣誉。扎实推进“平安贺州”建设,推行复合型警务机制改革,组织开展“春季攻势”专项行动、社会治安综合整治“三大战役”和严打整治行动,深入开展“大接访、大排查、大调解、大防控”活动,公众安全感和满意度明显提高。

　　 （九）着力抓好自身建设。开展“强作风、重落实、促发展”活动,推动21项重点工作有效落实。加强和改进政府法制建设,依法行政水平和质量有新的提高。坚持实行重大事项向市委报告制度,市政府重大决策邀请市人大、政协领导参与讨论,定期向市人大报告、向市政协通报工作。人大代表建议和政协委员提案办复率100%。加大政府信息公开力度。严肃换届纪律,深入推进党风廉政建设责任制,加强审计监督和行政监察。查办各种违法违纪案件135件。“三公”经费支出不超过经费预算规模。清理行政审批事项618项,办理审批项目提速78.4%,群众满意率达100%。加强与各民主党派、无党派人士、工商联和工会、共青团、妇联的联系。统计、工商、盐务、烟草、邮政、通信、气象、水文、老龄、残联、档案、地方志、机构编制、应急管理、行政服务、决策咨询、驻梧管理、住房公积金、供销合作、二轻联社、质量监督、检验检疫、防震减灾、库区移民、外事侨务、民族宗教等工作都取得了新的成绩。

　　 一年来,我们全面做好政府工作有以下几点体会:必须加快转方式调结构步伐,不断提高可持续发展水平；必须加快引进大企业大集团,高度重视中小企业发展；必须在不断加大自身投入的同时,积极争取国家各方面支持；必须既注重克服近期困难、解决突出矛盾,又重视为长远发展打好基础；必须切实保障和改善民生,以实实在在的发展成果惠及群众；必须坚持齐心协力狠抓工作落实,把社会各界力量汇聚成加快发展的巨大动力。

　　 各位代表,我们取得的成绩来之不易。这是全市人民共同努力的结果,也是社会各界关心支持的结果。在此,我代表市人民政府,向辛勤工作在各条战线的广大干部群众,向给予政府工作大力支持的人大代表和政协委员,向驻贺部队、武警官兵和政法干警,向各民主党派、工商联、人民团体和社会各界人士,致以崇高的敬意和衷心的感谢!

　　 在看到成绩的同时,我们也清醒认识到发展中遇到的困难和问题:我市经济总量偏小现状还没有根本改变,二、三产业有待进一步提升；融资难、土地供给不足、环境约束趋紧,制约着项目建设快速推进；电力供应紧张,节能降耗与经济增长矛盾突出,对工业发展造成较大影响；群众收入提高不快,物价仍然偏高,保障和改善民生压力还很大；社会群体利益诉求多样化,社会管理需要在创新中不断加强等。对于这些问题,我们将高度重视,认真加以解决。

二、2012年工作任务

　　 今年是实施“十二五”规划承上启下的关键之年,党的十八大将胜利召开,贺州也将迎来建市十周年大庆,做好各项工作,具有特殊而重大的意义。我们必须切实把思想和行动高度统一到中央和自治区党委、政府以及市委对形势的科学判断和决策部署上来,确保实现更好更快发展目标。一方面,要认识和把握有利机遇。当前,世界和平发展主题没有变、经济全球化大趋势没有变、结构调整大环境没有变；我国经济已经朝着宏观调控的预期方向发展,今年又实施积极的财政政策和稳健的货币政策,宏观政策环境将趋于有利。国家深入实施新一轮西部大开发战略,全面启动新十年扶贫开发工作；自治区加快建设桂东国家级承接产业转移示范区,大力支持贺州华润循环经济示范区建设,深化与广东的战略合作,明确支持新建市加快发展,又适逢我市建市十周年,发展扶持政策对我们加快基础设施、产业、民生项目建设十分有利。随着大通道网络不断完善,支撑我市加快发展的基础条件显著增强；现阶段我市正迈向工业化中期阶段,进入推进城镇化进程的黄金时期,大型央企、区企和民企、外企纷纷进驻贺州,一批重大项目陆续开工建设,经济增长的内生动力不断增强。另一方面,要正视和化解不利因素。受欧美债务危机等冲击影响,世界经济形势依然复杂多变,不稳定不确定因素明显增加。我国经济下行和通胀压力上升并存,宏观调控面临更多的“两难”选择。广西也面临经济增速风险大、控制物价上涨和节能减排压力大、保障和改善民生难度大等问题,以及各种生产要素供给不足等难题。以上我国我区发展存在的问题和难题在我市都不同程度地存在,有些方面表现得更为明显,特别是融资、用地、用电需求和供应矛盾仍十分突出,将给贺州加快发展带来诸多不利影响。对此,我们要把困难估计得更充分一些,把应对措施准备得更周全一些,努力保持和扩大经济社会发展的良好态势。

　　 2012年政府工作的总体要求是:全面贯彻中央及全区经济工作会议精神,以邓小平理论和“三个代表”重要思想为指导,深入贯彻落实科学发展观,紧紧围绕市第三次党代会提出的“两年有新突破、五年上新台阶、十年实现新跨越”的发展目标,按照“稳中求进、进中求快、快中保好”的主基调,深入实施“工业立贺、富民强市”战略,不断调整优化经济结构,加快新型工业化、城镇化、农业现代化步伐,深化改革开放合作,加强生态文明建设,保障和改善民生,创新社会管理,推进“五城联创”,正确处理好总量增长与质量提升、经济发展与改善民生、改革发展与维护稳定的关系,加快建设崛起的崭新贺州。

　　 2012年经济社会发展的主要预期目标是:地区生产总值增长13%,财政收入增长18%,全社会固定资产投资增长20%,规模以上工业总产值增长30%,规模以上工业增加值增长22%,社会消费品零售总额增长18%,外贸进出口总额增长15%；城镇居民人均可支配收入增长11%,农民人均纯收入增长12%；城镇登记失业率控制在4.5%以内,人口自然增长率控制在8.5‰以内；居民消费价格指数增幅控制在4.5%左右；节能减排指标控制在自治区下达的“十二五”目标年均增幅范围内。

　　 围绕上述目标,今年要重点加快以下十一个方面工作:

（一）加快推进新型工业化,推动产业优化升级

　　 加快工业园区和工业走廊建设。以桂东承接产业转移示范区上升为国家战略为契机,统筹工业园区规划建设管理,加大引企入园力度,集中力量推进园区基础设施和产业项目建设。确保贺州华润循环经济示范区火电厂、啤酒项目年内同步建成投产,示范区核心区建成并投入运行。确保电子科技生态产业园一期基础设施及进厂道路竣工,桂东电子、桂海铝业、江浩电子等一批铝电子产业项目开工建设。加强与中国铝业集团、中国有色矿业集团合作,加快旺高工业区内稀土产业园、钨钛锡新材料产业园建设步伐。加强与中国通用技术集团合作,开发利用丰富的大理石资源,着力把西湾(平桂)工业区建成全国重要的石材粉体深加工基地。加快两广合作信都经济区规划建设步伐,积极推动信都经济区上升为自治区层面发展战略,建设成为承接东部产业转移的桥头堡。加快工业园区连线建设,实现望高至富川二级路改造项目竣工通车,推进贺州至富川一级公路建设,使百里工业走廊成为承接产业转移的主战场。

　　 加快发展百亿元产业。确保工业投资额增长25%以上,推动电力、林产、矿业、电子、新材料等五大支柱产业加快发展,占全市规模以上工业总产值比例提高到80%以上,并力争经过2-3年的加速发展,形成百亿元产业集群,增强对经济发展的支撑能力。加快推进华润贺州电厂建设,加强与南方、华中等大电网合作,不断扩大桂东电力的产业优势,构建完善的区域供电网络。加强与中国纸业集团对接,推进华桂林桨纸一体化项目建设,加快贺达纸业重组改造工作,实现欣荣星中高密度纤维板等项目如期竣工投产,完善林产林化产业链。充分发挥市矿业投资公司作用,进一步整顿和规范矿产资源开发秩序,做大做强矿业经济。加快建设广西电子铝产品质量监督检验中心,抓好桂东电子、吉光电子迁建扩能项目建设,推进翔隆电子、玖典电子等项目建设,精心打造铝电子产业集群。开工建设平桂飞碟钨系列产品深加工项目,加快金源稀土、金广稀土提升扩能工作,促进金利新材料项目实现开工,做强做大钨钛锡和稀土功能材料等新材料产业。

　　 大力实施“抓大壮小扶微”工程。注重加强企业扶持管理,通过产业引导、政策配套、兼并重组、环境优化、培训服务等措施,着力形成各类经济竞相发展的良好局面。力争新增亿元企业10家,新增规模以上企业20家。推进中小企业自主创新,鼓励企业采用高新技术和先进适用技术改造提升传统产业,确保完成技术改造投资125亿元。全面落实国家及自治区鼓励民营经济加快发展的政策措施,鼓励民营资本参与各领域建设。实施微型企业创业扶持管理办法,积极鼓励支持全民创业,年内扶持发展微型企业600家。加强生产要素供给协调,重点保障全市工业56亿千瓦时用电需求,提高企业经营效益和市场竞争力。确保工业总产值突破500亿元,工业增加值达到170亿元以上。

　　 （二）加快推进新型城镇化,倾力打造城乡新面貌

　　 狠抓城市建设管理。举全市之力推进城建、交通、社会事业等六大类60个市庆项目建设。重点推进城东新区、江南新区、平桂新城等新城区建设,加快太白湖公园、贺州大酒店、灵峰大桥等标志性工程建设,完善城区路网、文化教育、医疗卫生等公共服务设施,加快形成“一江两岸三新区”发展新格局。开展创建国家森林城、文化先进城、全国双拥模范城、广西卫生城、社会和谐稳定模范城“五城联创”活动,全面提升城市综合竞争力。确保城镇化率提高到38%。

　 　提高城镇规划建设水平。完善城镇体系规划,进一步明确城镇空间布局和规模,加快城镇控制性详细规划编制,引导镇村合理布局和适度集中建设。深入实施城乡风貌改造工程,抓好广西特色名镇名村建设。积极探索各工业园区和旅游景区与所在乡镇之间的基础设施共享机制,重点加快建设以贺州华润循环经济示范区、旺高工业区、信都经济区、黄姚综合旅游示范区为依托的城镇群。

　　 大力发展县域经济。以推进扩权强县为契机,打造特色鲜明、主业突出、竞争力强的经济强县。八步区侧重抓承接产业转移、特色农业深加工、商贸物流业；钟山县侧重发展壮大机械装备、特色农业产业；富川瑶族自治县侧重发展循环工业、特色生态农业和瑶族文化旅游；昭平县侧重构筑林产、矿业为支柱的现代产业体系,进一步做大旅游产业和特色生态农业；平桂管理区侧重发展战略性新兴产业。

　　 （三）加快推进重点项目建设,拉动经济稳步增长

　　 抓好项目策划。把握国家加大农业、水利、民生、文化、社会事业等领域投入力度的有利时机,以基础设施、产业发展、民生项目为重点,加强项目库建设。坚持新开工和续建项目并举,落实一批1000万元以上的重大项目进行重点管理,形成固定资产投资的稳定支撑,确保重点项目滚动推进。

　　 推动项目建设。争取将贺州精铝、桂东电子工业园区标准厂房、农机汽车配件及汽车物流园、客家文化休闲公园等一批项目列为自治区层面统筹推进重点项目,尽快实现项目落地建设。突出抓好在建和续建项目,力争雨润200万头生猪加工、宏城工业基地建筑陶瓷一期、大桂山林场50万立方米人造板生产线等一批重点项目竣工投产或部分投产。确保完成全社会固定资产投资550亿元以上。

　　 破解项目建设难题。继续实行市领导联系重点项目工作责任制,完善项目督查制度和绩效考核问责制度。优化重点项目审批“绿色通道”,提高项目申报、审批效率。抓好土地收储经营,推进城乡建设用地增减挂钩,完善征地拆迁政策机制,努力缓解供地不足问题。积极拓展银行信贷融资主渠道,充分发挥国有融资平台作用,有效缓解融资难问题。

　　 （四）加快发展现代农业,建设农村美好家园

　　 推进新农村建设。全面落实强农惠农富农政策,深入实施万元增收工程、“234”农业产业化工程和三大增粮增收行动,大力推进农业机械化水平,实现农机总动力增长10%,确保全年粮食种植面积稳定在206万亩,粮食产量稳定在70万吨,农林牧渔业增加值增长4.6%以上。坚持“以城带乡”,推进生态小康示范村建设。抓好农村规划、综合治理和产业发展,加强农村道路、电网、卫生室等公共服务设施建设,打造“十镇百村百里文明走廊”。

　 　大力发展特色优势产业。突出抓好瘦肉型猪、优质家禽等规模养殖发展,着力发展昭平茶叶、富川脐橙、钟山大头菜、平桂马蹄、八步蔬菜等现代特色种植业。加强原产地产品及地理标志产品保护工作。抓好281个生态农业示范村(屯)建设,深入推广以沼气为纽带的生态循环农业模式,实现技术覆盖面积55万亩。加快广西首个市级出口食品农产品质量安全示范区建设,健全完善农产品质量监管体系。

　　 加快农业产业化步伐。深入实施龙头企业带动战略,完善龙头企业与农户的利益联接机制,不断提高农业产业化组织程度,重点推进山茶油精深加工、肉制品冷冻加工、茶叶加工、果蔬保鲜加工、优质粮油加工。加快推进金泰农产品加工物流中心、富川特色农产品加工物流中心项目建设,打造区域性农产品物流集散中心。

　　 （五）加快发展现代服务业,努力扩大消费需求

　 　进一步提升商贸旅游业。加快桂台(贺州)客家文化旅游合作示范区建设,推进姑婆山客家风情小镇、钟山十里画廊、桂江生态旅游等项目建设。大力发展乡村旅游,拓展旅游客源市场,确保实现接待游客人数、旅游总收入、旅游创汇等三项指标均增长10%以上。做好物流体系规划建设,优化城市商业结构和空间布局,加快推进湖广大市场、市客运北站、旺高物流中心建设,启动交通商贸物流园、农机汽车配件及汽车物流园、无水港区物流中心等项目。

　　 大力发展各种服务业。推进“无线城市”、“数字贺州”建设,加快智能交通、安防监控、数字社区等物联网重大应用。提升酒店、餐饮等传统服务业,积极稳妥发展房地产业,发展信息咨询、商务中介、营销策划、产权交易、社区服务等新兴服务业。

　　 积极扩大消费需求。加强城乡市场流通体系建设,深入推进“万村千乡”市场工程和农贸市场改造升级。拓宽和开发消费领域,大力发展以脐橙、贡柑、茶叶等优势农产品和以住房、汽车等消费热点为载体的展会经济,促进居民文化、旅游、健身、养老、家政等新兴服务消费。坚决打击商业欺诈、制假售假行为,让广大群众方便消费、放心消费、安全消费。

　　 (六)加快改善发展环境,提高经济可持续发展水平

　　 推进交通项目建设。推进贺州机场、贺州至河池巴马高速公路和柳韶铁路贺州段等项目前期工作,加快贵广高速铁路贺州段、永贺高速公路贺州段、贺江扩能(改建)工程建设,加快构建“五高三铁两江一机场”立体交通框架。推进贺州火车站至西湾工业基地公路项目二期工程、贺州大道南出口至旺高工业区道路工程、贺州至姑婆山公路和江南路网、城东新区路网等项目建设,加大通乡油路、通村沥青(水泥)路等农村公路项目建设力度,全面提高市内交通密度、速度和通达程度。

　　 推进水利、能源基础设施建设。掀起冬春水利建设新高潮,深入实施农村饮水安全保障、中小河流治理、病险水库除险加固和农田水利建设“四大工程”,做好贺江治理工程、路花水库水源工程等重大项目前期工作,加强城区、钟山县城、昭平县城等防洪堤建设,抓好城区狮子岗、黄安寺等排洪河治理,推进龟石水库等水源地水生态系统保护与修复建设。推进上程电站和富川龙头、长春、金子岭风电项目等电源建设,加强桂东电网、信都二、贺街等220千伏输变电项目建设,抓好农网升级改造和村村通电建设工程。加快城市天然气管网建设,尽快建成燃气供应系统。

　　 加强生态文明建设。深入实施“绿满贺州”工程,确保完成造林绿化23万亩。深入实施山区生态林、重点区域防护林、自然保护区建设和小流域水土保持综合治理等重点生态工程。全面实施“十二五”节能减排综合性工作方案,严控重点能耗企业电耗增长量,强化对市、县两级污水处理厂、季节性生产企业监管力度。

　　（七）加快改革开放步伐,开创对外开放合作新局面

　　全面推进各项改革。继续深化农村改革,积极推进土地承包经营权流转和集体林权制度改革。推动工业园区行政管理、农业事务、城建管理等体制改革,支持平桂管理区创造条件申报市辖区工作。健全以合同管理为基础的事业单位用人机制,深化事业单位收入分配制度改革。

　　加强区域交流合作。深化与梧州、玉林、贵港三市的务实合作,积极争取国家、自治区各方面支持,把桂东承接产业转移示范区建设成为西江经济带核心发展区域。深化与广东佛山、清远、韶关等市际交流合作,以电子科技生态产业园、西湾(平桂)工业区为重点,抓好“飞地经济”园区规划建设,在新一轮产业结构调整中抢占承接产业转移先机。加强与央企、知名民营企业和自治区重点企业合作,推动重点领域开放开发。确保内资项目到位资金增长15%；外资项目到位资金增长20%。

　　扎实推进对外贸易。以市场为导向,培育新的出口增长点,扩大特色优质农产品、工业产品的出口份额。积极组团参加各类国内外展会活动,充分利用桂台经贸合作交流会、中国-东盟博览会等交流平台,开拓出口产品的国际市场。

　　 （八）加快推进财政金融工作,增强发展保障能力

　　 加强税源培植。继续发挥财政资金引导作用,综合运用贴息、补助等政策手段,加大对支柱产业、重点企业和重点项目的扶持力度,打造新的税源载体,确保财政收入总量、质量得到“双提升”。抓好国有资产经营、经营城市和土地管理工作,夯实财政稳定增长基础。

　　 强化财政收支管理。加强对重点税源企业、重大建设项目跟踪管理,确保税收及时、足额、均衡入库。深化“收支两条线”改革,确保各项非税收入全部纳入预算管理。加大征收稽查监督力度,完善“以电控税”、“电子计量”管理办法,确保税收收入占一般预算收入比重稳步提高。积极调整优化支出结构,优先解决中央投资项目地方配套资金、重点项目建设、保障和改善民生等领域的财政支持。

　　 做好金融保险工作。深化政企银合作,推动金融支持经济社会发展政策措施落实,引导金融机构加大对城市建设项目、农田水利基础设施建设、中小企业和农户信贷的投放力度,力促已授信贷款顺畅发放。积极推进农村金融改革试点,大力发展村镇银行和租赁、担保、小额贷款等新型金融机构,进一步完善和扩大政策性农业保险试点工作。

　　 （九）加快发展文化事业,增强文化软实力

　 　提升社会文明水平。加强中华优秀传统文化教育,广泛组织城乡开展社区演出、文化下村、主题广场晚会、城乡文艺汇演等各类文化活动,培育和塑造贺州精神,深入开展群众性精神文明创建活动。

　 　完善文化基础设施。做好市图书馆、群众艺术馆、博物馆、档案馆等项目前期工作,加快市文化中心、传媒中心建设。实行市县两级文化馆、图书馆、博物馆免费开放,建设完善乡镇综合文化站、村级公共服务中心、农家书屋等硬件设施。加强基层广播影视基础设施建设,深入推进20户以下广播电视“村村通”工程建设。

　 　加快发展文化产业。实施文化人才“十百千”培养工程,培养造就一批民族、民间优秀文化传播者、当代文化艺术家和文化企业家。大力挖掘麒麟尊文化、茶文化、莲文化、奇石文化、长寿文化等地方特色文化,创作一批文艺精品。深入挖掘客家文化、瑶族文化等资源,提高客家山歌剧、瑶族歌舞等创作水平,打造“瑶族盘王节”、“客家文化旅游节”等特色文化品牌。

　 　（十）加快实施民生工程,促进社会和谐进步

　 　注重保障和改善民生。继续实施为民办实事工程。强化社会保险扩面征缴,完善失业、工伤保险和城乡最低社会保障制度,积极推进城镇职工生育保险市级统筹工作,加快实现养老保障体系城乡全面覆盖,城镇居民医保参保率稳定在96%以上,新农合参保率达到95%以上。提高农村五保对象生活保障水平。全面落实就业有关政策,构建和谐劳动关系,重点做好零就业家庭、就业困难人群的就业再就业工作,全年实现就业再就业1.6万人。继续实施整村推进贫困村扶贫开发,解决1.8万贫困村群众稳定增收难题。加快建设多层次保障性住房体系,年内完成8900套保障性安居工程目标任务,推进驻梧管理处大院危旧房改造,加快农民拆迁回建房建设。加快城乡救助体系建设,做好双拥优抚安置工作。综合运用多种调控手段,全面加强价格调控监管,建立健全调控和应急保供预案,保持物价总体水平基本稳定。

　 　加快发展各项社会事业。扎实推进第五轮创新计划,全面实施知识产权战略,培育发展高新技术产业。加快推进中心城区教育项目建设大会战,重点新建、扩建贺州高级中学等8所中小学校,全面完成中小学校舍安全工程三年规划建设任务。积极推进医药卫生体制改革,深入实施防治艾滋病攻坚工程。加快市人民医院医技综合楼、市精神病医院,以及八步区、平桂管理区人民医院等项目建设,强化乡镇卫生院、村卫生室等农村基层医疗卫生基础设施建设。促进群众体育和竞技体育、体育设施和体育产业协调发展。深化人口计生综合改革,创新开展“诚信计生”,积极构建“幸福计生”。完善利益导向机制,稳定低生育水平。

　　 做好其他工作。充分发挥工会、共青团、妇联等人民团体和社会组织参与管理、服务的重要作用,认真落实党的民族、宗教和侨务政策,进一步做好统计、烟草、气象、老龄、残联、地方志、机构编制、驻梧管理、住房公积金、供销合作、二轻联社、质量监督、检验检疫、库区移民、防震减灾等方面工作。

　　（十一）加快转变职能,建设人民满意的服务型政府

　 　提高社会管理水平。努力建设具有贺州特点的社会管理体系。深化“平安贺州”建设,推进“天网工程”三期项目实施,集中整治重点地区和突出问题,完善社会治安综合防控体系。建立健全乡镇、村、屯三级信访维稳工作网络,加大基层矛盾纠纷调解排查力度。深入开展食品药品安全专项整治,严厉打击制售假劣食品药品违法行为。严格落实安全生产责任制和责任追究制。完善市、县、乡三级政府应急体系建设,增强应对公共突发事件处置能力。

　 　增强执行力和公信力。加快推进依法行政和建设法治政府。完善重大事项集体决策、专家咨询、社会公示和听证制度,落实人大代表议政制度和政协委员协商制度,广泛听取各民主党派、无党派人士、工商联意见,健全社情民意反馈制度,使各项政策更加符合实际,经得起考验。加强对重点工作执行情况的核查监督,做到令行禁止。扎实开展政务服务、政务公开、政府信息公开工作,年内完成所有乡镇建立政务服务中心任务。

　 　高度重视反腐倡廉建设。建立健全预防腐败体系各项制度,加强对领导机关和领导干部、重点环节和重点部位的监督。强化审计监督和行政监察,把政府采购招投标等社会公共资源项目纳入自治区三级联网电子监察系统。严格控制会议、公务接待、公费出国、公务购车用车等经费支出。严肃查处各种违法违纪案件。

　 　各位代表!科学发展谱新篇,而今跨越正其时。让我们在自治区党委、政府和市委的坚强领导下,继续紧密团结依靠全市各族人民,克难攻坚,开拓进取,扎实工作,全面完成全年的各项工作任务,以优异的成绩迎接党的十八大胜利召开和建市十周年,为加快建设富裕文明和谐幸福新贺州而努力奋斗!
